# Ultrafiltration Fractionation of Bovine Hemoglobin Hydrolysates: Prediction of Separation Performances for Optimal Enrichment in Antimicrobial Peptide

**DOI:** 10.3390/membranes11020073

**Published:** 2021-01-20

**Authors:** Sophie Beaubier, Rémi Przybylski, Alice Bodin, Naïma Nedjar, Pascal Dhulster, Romain Kapel

**Affiliations:** 1 Laboratoire Réactions et Génie des Procédés, Université de Lorraine, UMR CNRS 7274, LRGP, F-54500 Vandœuvre-lès-Nancy, France; sophie.beaubier@univ-lorraine.fr (S.B.); bodin.alice@gmail.com (A.B.); 2 EA 7394—ICV—Charles Viollette Institute, University Lille, INRA, ISA, University Artois, University Littoral Côte d’Opale, F-59000 Lille, France; remi.przybylski@polytech-lille.fr (R.P.); naima.nedjar@univ-lille.fr (N.N.); pascal.dhulster@univ-lille.fr (P.D.)

**Keywords:** antimicrobial peptide, ultrafiltration, peptide separation, simulation, valorization

## Abstract

Hydrolysis of bovine hemoglobin (bHb), the main constituent of bovine cruor by-product, releases a natural antimicrobial peptide (NKT) which could present a major interest for food safety. To enrich this, tangential ultrafiltration can be implemented, but ultrafiltration conditions are mainly empirically established. In this context, the application of a simulation method for predicting the NKT yield and enrichment was investigated. Ultrafiltration performances were studied for decolored bHb hydrolysates at different degrees of hydrolysis (DH; 3%, 5%, 10% and 18%) and colored hydrolysates (3% and 5% DH) with 1 and 3 kg·mol^−1^ regenerated cellulose membranes. The simulation method helped to identify the most promising hydrolysate (in terms of NKT enrichment, yield and productivity) as the 3% DH colored hydrolysate, and UF conditions (volumetric reduction factor of 5 and 3 with 1 and 3 kg·mol^−1^ membrane, respectively) for higher antimicrobial recovery. A maximal enrichment factor of about 29 and NKT purity of 70% in permeate were observed. The results showed that the antimicrobial activity was in relation with the process selectivity and NKT purity. Finally, this reliable method, applied for predicting the ultrafiltration performances to enrich peptides of interest, is part of a global approach to rationally valorize protein resources from various by-products.

## 1. Introduction

In the food industry, bovine blood is an inevitable part of meat production and involves an environmental problem due its lack of harnessing [1]. Blood is rich in hemoglobin, a protein widely described as an excellent and emerging source of antimicrobial peptides [2]. One of them is particularly interesting in comparison with other antimicrobial peptides by means of specific features. Indeed, the α137-141 peptide (also called neokyotorphin or NKT) is a small peptide (653 Da, pI 10.5) composed of five amino-acids (Thr-Ser-Lys-Tyr-Arg), while most antimicrobial peptides are composed of more than 20 amino acids for a molecular weight higher than 1500 Da [3]. Moreover, NKT contains no hydrophobic residue, involving a particular and strong antimicrobial mechanism with no secondary structure unlike most antimicrobial peptides [4]. This peptide was previously described as a growth inhibitor of pathogenic bacteria commonly involved in the contamination phenomenon during the storage and distribution of food [5]. More recently, NKT derived from bovine hemoglobin showed antimicrobial and antioxidant properties as effective as the BHT activities after application into ground beef meat under refrigeration [6]. Therefore, this peptide could be a promising natural preservative. From this previous study, NKT appeared early during the pepsic hydrolysis of bovine hemoglobin (bHb) and it was a final peptide, i.e., not cleaved into a smaller peptide. Indeed, its production can reach about 100 mg. L^−1^ after 24 h, with a decreasing production rate after 30 min when more than 67% of NKT has been produced. Peptide enrichment from bHb could be influenced by the presence of the haem, responsible for the red color of bHb [7]. Haem also showed an increase in the bitterness of the final product and strong oxidative capacities which had a negative impact on the preservation of the hydrolysate and its bioactivity [8]. Consequently, some processes were developed to remove haem while the peptides were recovered and maintained in solution [9,10]. However, increasing the purity of the peptide is the key to obtaining better activity for a potential and attractive market product [11].

Tangential ultrafiltration is well known to improve a large spectrum of hydrolysate bioactivities because of bioactive peptide enrichments [12,13,14,15,16,17,18]. However, in the studies reported, the ultrafiltration (UF) conditions (membrane molecular weight cut-offs (MWCO), transmembrane pressure, hydrolysate concentration, volume reduction factor, tangential velocity, etc.) were empirically established after many runs of experiments implying fastidious procedures for the assessment of bioactivity. Some authors showed that the yield and enrichment of micropeptide fractions could be monitored during UF by analytical size exclusion chromatography (SEC) [19,20]. Based on these results, Kapel et al. [21] proposed a simulation method to predict the yield and enrichment of a targeted bioactive peptide in a UF permeate compartment. The simulation method, based on solute mass balance equations, only requires a SEC chromatogram and the protein aminogram. This method was validated with success with an alfalfa white protein hydrolysate, containing an antihypertensive peptide in a single UF operating condition.

In this article, the simulation method was applied to predict the performances of UF for the NKT yield and enrichment of decolored bHb hydrolysates at different degrees of hydrolysis (3%, 5%, 10% and 18% DH) and in colored hydrolysates (3% and 5%) with 1 and 3 kg·mol^−1^ regenerated cellulose membranes. Then, the most promising trade-offs in terms of NKT enrichment, yield and productivity were implemented and the antimicrobial activities of the fractions obtained were evaluated in four microorganisms known to cause food safety problems (*Micrococcus luteus*, *Listeria innocua*, *Escherichia coli* and *Salmonella enteritidis*). Eventually, the antimicrobial activities versus the NKT purity of the fractions were discussed.

## 2. Materials and Methods

### 2.1. Material

HPLC grade acetonitrile was purchased from Fisher Scientific (Waltham, MA, USA). Trifluoroacetic acid was obtained from Arcos Organics (Waltham, MA, USA). Sodium hydroxide pellets were supplied by Carlo Erba Reactifs (Val de Reuil, France). Bovine hemoglobin (bHb) and porcine pepsin were from Sigma Aldrich (Saint Quentin Fallavier, France). The mean molar mass by amino acid $\bar{MW_{aa}}$ and the mean molar extinction coefficient by amino acid $\bar{\varepsilon_{aa}}$ of the bHb was calculated from its primary sequence as 115 g·mol^−1^ and 987 L·mol^−1^·cm^−1^, respectively.

### 2.2. Hydrolysate Preparation

A stock solution was prepared by adding dried bHb (Sigma Aldrich, Saint Quentin Fallavier, France) into 100 mL of ultrapure water and the precise bHb concentration was measured by the Drabkin method [22] after centrifugation (30 min, 4000 tr·min^−1^) to remove the insoluble part. The stock solution was diluted to obtain the required concentration of 1% (*w*/*v*). Hydrolysis was carried out at pH 3.5 and under constant temperature (23 °C), using pepsin from porcine gastric mucosa (EC 3.4.23.1, 3200–4500 units.mg^−1^ protein) with an enzyme/substrate ratio of 1/11 (mole/mole). The hydrolyses were stopped by adding sodium hydroxide (5 M) up to a final pH of 9.0 after 2.5 and 10 min, 3 and 24 h corresponding to respective degrees of hydrolysis (DH) of 3, 5, 10 and 18% [6], assessed by the ortho-phthaldialdehyde method [23]. These hydrolysates were identified as “whole hydrolysates”, called “colored hydrolysates”. A previous study showed that the presence of haem has a negative impact on the peptide transfer during the separation process using an ultrafiltration membrane [7]. The removal of haem consisted of slowly lowering the pH to a value of 4.7 with hydrochloric acid (2 M at 2 mL·min^−1^). After 24 h at room temperature, the haem was totally precipitated, and the peptides were maintained in solution. Both phases were separated by centrifugation (30 min, 4000 tr·min^−1^). In this paper, the hydrolysates without haem were called “decolored hydrolysates”.

### 2.3. Yield and Enrichment Simulation Methodology

#### 2.3.1. Membrane Calibration

The simulation of the evolution of the yield and enrichment during the UF is based on a membrane calibration equation [21]. This calibration was achieved by analyzing the UF permeate and retentate outlets in total recirculation mode by size exclusion chromatography (SEC). A value of retention (R_x_) was calculated for each point “x” of the profile, as follows

(1) $$R_{x}\;=\;1-\;\frac{A_{p,x}}{A_{r,x}}$$

with A_p,x_ and A_r,x_ as the absorbances of the point eluted at the time “x” in permeate and retentate, respectively. Then, a molar weight value was associated based on its elution time and the SEC column calibration. The membrane calibration equation was yielded by linear regression of (R vs. log (MW)) applied to each point “x”. The 1 and 3 kg·mol^−1^ membrane calibrations were achieved for each hydrolysate considered in the study at 3 bar of transmembrane pressure (TMP), a concentration of hydrolysate of 10 g.L^−1^ and a retentate flow rate of 0.1 L·min^−1^.

#### 2.3.2. Yield

In UF, the yield (*η*) of a solute “i” in the permeate compartment was given by Equation (2)

(2) $$\eta =\frac{\bar{C_{p,i}}}{C_{0,i}}\;\times \left(1-\frac{1}{\mathrm{VRF}}\right)$$

with $\bar{C_{p,i}}$ and $C_{0,i}$ as the concentrations of the solute “i” in the permeate compartment and in the starting hydrolysate, respectively, and the volume reduction factor (VRF) calculated as VRF = V_0_/V_R_, with V_0_ and V_R_ being the initial volume and the volume in the retentate compartment.

The relative concentration of “i” can be deduced from the mass balance as follows:

(3) $$\frac{\bar{C_{p,i}}}{C_{0,i}}=\frac{{\mathrm{VRF}-\mathrm{VRF}}^{R_{i}}}{\mathrm{VRF}-1}$$

The retentions of NKT (MW = 653 g·mol^−1^) with the 1 and 3 kg·mol^−1^ membranes were 0.625 and 0.305, respectively. These values were deduced from the membrane calibration equations obtained with the bHb decolored hydrolysate at 10% DH (R = 0.7978 × log (MW)—1.621, with 1 kg·mol^−1^ membrane, and R = 1.055 × log (MW)—2.666; with 3 kg·mol^−1^ membrane).

#### 2.3.3. Enrichment

The enrichment (τ) of a solute “i” in the UF permeate compartment was given by Equation (4)

(4) $$\tau =\frac{\bar{C_{p,i}}}{C_{0,i}}\times \frac{\sum^{\;}C_{0}}{\sum^{\;}\bar{C_{p}}}$$

with $\sum^{\;}C_{0}$ and $\sum^{\;}\bar{C_{p}}$, the peptide concentrations of the starting hydrolysate and the permeate UF fraction, respectively.

The peptide concentrations in the permeate compartment $\bar{C_{p,i}}$ can theoretically be calculated for a given VRF applying Equation (3) to each peptide comprising the hydrolysate. This supposes knowledge of every peptide molar weight and concentration in the hydrolysate, which is impossible for such complex mixtures. The originality of the simulation methodology consists of assessing the permeate peptide concentration from the hydrolysate SE-chromatogram. First, the UV signal of the chromatogram was converted into a concentration according to Kapel et al. [21]. Then, Equation (3) was applied to each point “x” of the chromatogram to give a calculated permeate chromatogram. The retention R value used for each chromatogram point was deduced from the elution time using the SE column and the membrane calibration. Eventually, this permeate chromatogram was integrated to give $\sum^{\;}\bar{C_{p}}$ at any VRF.

### 2.4. Ultrafiltration Experiments

The ultrafiltration experiments were performed with regenerated cellulose UF membranes (MWCO of 1 and 3 kg·mol^−1^ with a surface of 88 cm^2^, Millipore, Burlington, Massachusetts, USA). The experiments were conducted on a Cogent µScale TFF system (Millipore) at room temperature (22 °C +/− 2 °C) and 3 bar of transmembrane pressure (under the critical TMP). The initial hydrolysate volume and concentration were 0.2 L and 10 g.L^−1^, respectively. The recirculation flow rate was 0.1 L·min^−1^. The permeate was collected after 20 min of total recycling (permeate and retentate outlets) to stabilize the polarization layer and the permeate flux. The UF experiments were stopped at a volume factor reduction (VRF) of 5. The experiments were performed in triplicate for both operating modes. The membranes were washed and stored in 0.1 M NaOH (storage at 4 °C).

### 2.5. Hydrolysates and Fraction Reverse Phase HPLC Analyses

The liquid chromatographic system consisted of a Waters 600E automated gradient controller pump module, a Waters Wisp 717 automatic sampling device and a Waters 996 photodiode array detector (Milford, USA). The methodology applied for the analysis and the peptide quantification was described in a previous paper [6]. Briefly, a standard curve was established by injections of standard NKT at concentrations ranging between 0 and 1 mg·mL^−1^. The following Equation (5) was used to link the area under the peak of NKT from the chromatographic profile at 214 nm to its concentration in the sample

C_NKT_ = 4419.6 × A_NKT_ (5)

with C_NKT_ being the NKT concentration (mg.L^−1^) and A_NKT_ being the area peak (µV.s).

The NKT purity (%) was assessed as follows

(6) $$\mathrm{NKT}\;\mathrm{purity}\;\left(\%\right)=\frac{A_{\mathrm{NKT}}}{A_{\mathrm{total}}}\times 100$$

with A_NKT_ being the area peak (µV.s) and A_total_ being the total peptide area of the chromatogram (µV.s).

### 2.6. Hydrolysates and Fraction SE-HPLC Analysis

The bHb hydrolysates and the UF fractions were analysed by SEC using a Superdex peptide HR 10/300 column (10 × 300 mm, GE Healthcare, Chicago, Illinois, USA) connected to a Shimadzu model LC20 system (Shimadzu Corporation, Kyoto, Japan). 50 µL of sample were injected onto the column. The mobile phase consisted in water and acetonitrile in a 69.9/30 proportion (*v*/*v*) with 0.1% TFA (*v*/*v*). Samples were eluted at a flow rate of 0.5 mL·min^−1^ and under a temperature of 35 °C. The UV signal was monitored at 214 nm using cells with an optical path of 1 cm. The column was calibrated with 45 synthesized peptides from 220 to 1890 g·mol^−1^ eluted in the same conditions (the calibration equation was log (MW) = −0.082.Tr + 5.382, with Tr, the elution time in min). The chromatograms were exported in Excel spreadsheets and the absorbance signals were converted into concentrations using the methodology reported by Bodin et al. [24].

The chromatograms in concentrations were integrated to quantify the peptide concentrations in the UF permeate compartments and calculate the peptide molar weight cumulative frequency (in mass). The productivity was also calculated from the total peptide concentration in the UF permeate compartment. The membrane surface area used (m^2^) and the ultrafiltration duration (h) were considered, and the productivity was expressed in g·m^−2^·h^−1^.

### 2.7. Neokyotorphin Relative Concentration Quantification by RP-HPLC/MS

The experimental C_p_/C_0_ values of NKT were determined by RP-HPLC/MS. The column used was a C18 Prosphere (250 × 21 mm, 5 µm diameter beads) provided by Alltech (Carquefou, France) connected to the Shimadzu model LC20 system. For the analysis, the column was kept at 35 °C. A total of 10 µL of sample was injected. For the elution, gradient of solvent A (water/acetonitrile in 94.9/5 proportion (*v*/*v*) with 0.1% TFA (*v*/*v*)) and solvent B (water/acetonitrile in 4.9/95 proportion (*v*/*v*) with 0.1% TFA (*v*/*v*)) was used. The starting condition was 100% A. A first slope was applied to reach 28% B/72% A in 50 min. Then, a second slope was applied to reach 48% B/52% A in 20 min. The column was finally washed with 100% B for 10 min and re-equilibrated in 100% A for 15 min. The flowrate was 0.2 mL·min^−1^. The eluent was analysed on-line by electrospray ionization mass spectrometry (ESI-MS) (Shimadzu Corporation, Kyoto, Japan) in positive mode. The following operating parameters were used in TIC mode: mass scan = 50–2000 m/z, ion spray tension = 4.5 kV, heat block temperature = 200 °C, drying gas flow = 15 L/min, scan speed = 2143 mass units/s. Selected Ion Monitoring (SIM) mode was also used to monitor the targeted peptides. The integration values of NKT MS signal were integrated to assess the C/C_0_ values in the UF samples.

### 2.8. Evaluation of Antimicrobial Activity

The antimicrobial activity was determined by measurement of the minimum inhibitory concentration (MIC) according to a previous study [25]. Briefly, the hydrolysates and the peptide fractions were tested against the growths of Gram-positive (*Micrococcus luteus* ATCC 4698 and *Listeria innocua* ATCC 33090) and Gram-negative bacteria (*Escherichia coli* ATCC 25922 and *Salmonella enteritidis* ATCC 13076), broadly described as inhibited by the bioactive peptides contained in the bHb hydrolysates [2]. A total of 20 µL of strain preculture was inoculated into 10 mL of liquid Lysogeny Broth (LB) medium and the preculture was performed on a rotary shaker (160 min^−1^) at 37 °C to obtain a standard cell concentration of 10^6^ CFU·mL^−1^ (colony-forming unit). A total of 100 µL of preculture was distributed in a microtiter plate well and added to 100 µL of the peptide fraction or hydrolysate. Each peptide sample was tested at least in triplicate.

The absorbance of each well was read at 630 nm against the blank obtained before incubation by using a microplate absorbance reader ELx808 with the Gen5 software (Biotek Instrument, Winooski, USA). MIC was the lowest peptide concentration that inhibited the strain growth after incubation (24 h, 37 °C) and was expressed in µg_peptide_·mL^−1^.

## 3. Results and Discussion

### 3.1. bHb Hydrolysates Characterization

**Figure 1:**
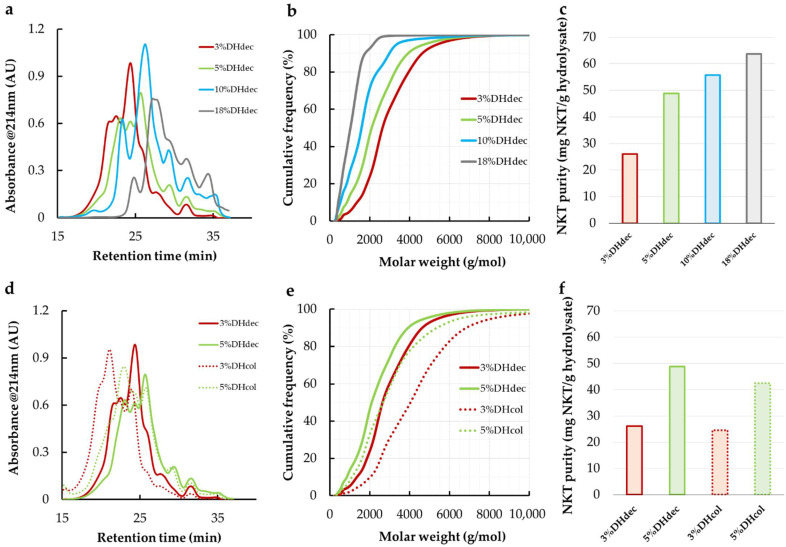
Size exclusion chromatograms at 214 nm of bovine hemoglobin hydrolysates (**a**,**d**); deduced peptide molar weights distributions (**b**,**e**) and natural antimicrobial peptide (NKT) purity (**c**,**f**). Letters (**a**–**c**) display data from 3%, 5%, 10% and 18% DH decolored (dec) hydrolysates while (**d**–**f**) compare 3% and 5% DH hydrolysates decolored or colored (col).

Figure 1a shows the SEC chromatograms of bHb hydrolysates at 3%, 5%, 10% and 18% DH after haem removal by acid precipitation (3%DH_dec_, 5%DH_dec_, 10%DH_dec_ and 18%DH_dec_). An increase in the elution volume range of peptides with increasing DH was observed. This indicated a decrease in the peptide molar weights during the proteolysis. Figure 1b shows that the median molar weight of the peptides decreased from 2600 to 1000 g·mol^−1^ from 3%DH_dec_ to 18%DH_dec_. This meant that the proteolysis followed a zipper-type mechanism. This is because, during the zipper-type proteolysis, the protein is fully hydrolyzed at the early stage of the reaction into a first set of intermediate peptides which are further hydrolyzed into final and smaller peptides at a higher degree of hydrolysis [26,27].

The neokyotorphin (NKT) purity in the hydrolysates increased from 26.1 mg NKT/g hydrolysate to 63.8 mg NKT/g in 3%DH_dec_ and 18%DH_dec_ (Figure 1c). The continuous increase in the NKT concentration during the proteolysis revealed that NKT is a final peptide produced at a relatively early stage of the proteolysis. This was also observed by others in a similar proteolysis condition [28]. Figure 1b showed that 3%DH_dec_ contained 2.7% (*w*/*w*) of peptides having a molar weight inferior to NKT (MW_NKT_ = 653 g·mol^−1^). This proportion raised up to 26.1% in 18%DH_dec_. Hence, despite the highest NKT purity, 18%DH_dec_ might not be the most appropriate hydrolysate for NKT enrichment by membrane separation.3% and 5% DH hydrolysates without haem removal (3%DH_col_ and 5%DH_col_) were also analyzed and compared to 3%DH_dec_ and 5% DH_dec_ (Figure 1d–f). The median peptide molar weight of 3%DH_col_ was 4000 g·mol^−1^ (against 2300 g·mol^−1^ in 3%DH_dec_). 5%DH_col_ also showed a higher median peptide molar weight than 5%DH_dec_ for an NKT purity very close to the corresponding decolorized hydrolysates (around 25 mg·g^−1^ at 3% DH and 45 mg·g^−1^ at 5% DH, Figure 1f). This is probably due to a loss of large-molar-weight peptides occurring during the decoloration procedure. Indeed, large peptides are known to be associated with haem at low DH by a complex system of peptide—peptide and peptide—haem interactions [10]. This resulted a large amount in peptides with a larger molecular size than NKT (99%) in 3%DH_col_ hydrolysate. Hence, an outstanding enrichment of NKT by ultrafiltration should be expected.

To summarize, the lower the DH was, the more hydrolysates are rich in large peptides, allowing for very high enrichment by UF. However, the higher the DH was, the higher the starting NKT purity was. Hence, each hydrolysate must be considered in the UF fractionation study for the NKT separation. From the hydrolysate molar weight distributions, it is reasonable to expect an interesting NKT purity in the permeate compartment of the 1 or 3 kg·mol^−1^ membranes.

### 3.2. NKT Enrichement by Ultrafiltration

A simulation methodology to predict the NKT enrichment and yield with the 1 and 3 kg·mol^−1^ membranes was used to avoid a time- and cost-consuming full experimental approach. This method only requires (i) a membrane calibration based on the SEC analysis of permeate and retentate in full recycling mode (both permeate and retentate outlets) and (ii) a SEC analysis of the hydrolysate [21].

#### 3.2.1. Membrane Calibrations

**Figure 2:**
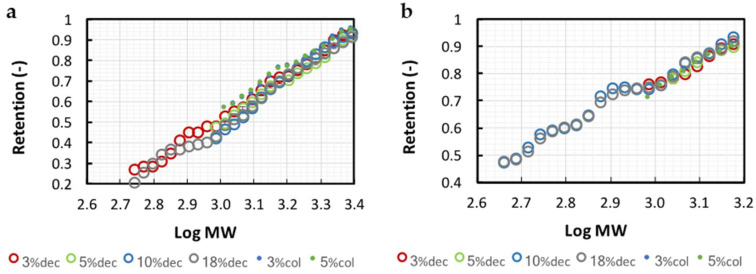
3 and 1 kg·mol^−1^ MWCO membrane calibration curves (respectively (**a**,**b**)) obtained with decolored 3%, 5%, 10% and 18% DH (dec) and colored 3% and 5% DH (col) hydrolysates. Retention versus log (MW) curves were built from permeate and retentate size exclusion chromatograms in total recirculation mode.

The calibrations of the 1 and 3 kg·mol^−1^ (MWCO) regenerated cellulose membranes obtained with each hydrolysate according to Kapel et al. [27] are presented in Figure 2. The calibration curves were reliably regressed by a linear model (0.995 > R^2^ > 0.988). Whatever the DH, the hydrolysates with or without haem showed very similar trends on both 1 and 3 kg·mol^−1^ MWCO membranes. This indicated that the peptide transport was mainly driven by phenomena related to steric hindrance. Hydrophobic interactions were shown to be responsible for peptide–membrane interactions [29] with PES and PS membranes. These interactions impacted peptide transmissions [30]. It is well known that the reduction in peptide size by proteolysis sensibly reduces the overall hydrophobicity pattern of the hydrolysate. Hence, a modification of the calibration should have been expected with the different DH hydrolysates. The poor effect observed here is probably due to the high hydrophilicity of the regenerated cellulose membranes surface [31] that reduces potential hydrophobic interactions with the membrane surface. The similarity observed between the different calibration curves showed that only one can be chosen for the following simulation calculations. This is particularly interesting because it shows that the membrane calibration obtained with a protein hydrolysate can be applied to any other hydrolysate from the same protein (with different DH). These results confirmed the theoretical reliability of this simulation methodology.

As expected, the peptide transmission was higher for the 3 kg·mol^−1^ membrane (retention of 0.5 for peptides of 1000 g·mol^−1^ against 0.78 with 1 kg·mol^−1^ membrane). The apparent MWCO (defined as the molar weight of a peptide retained at 90%) for bHb peptides differed from the given MWCO (1445 vs. 1000 g·mol^−1^ and 2510 vs. 3000 g·mol^−1^). Chabeaud et al. [20] also observed this phenomenon with a PES membrane (4 kg·mol^−1^) using a fish protein hydrolysate. This can be explained by the fact that manufacturers establish membranes MWCO based on standards with shapes that are different from peptides. NKT retention (R_NKT_) was of 0.305 and 0.652 with the 1 and 3 kg·mol^−1^ membrane, respectively.

#### 3.2.2. NKT Yield, Enrichment, and Purity Simulation

**Figure 3:**
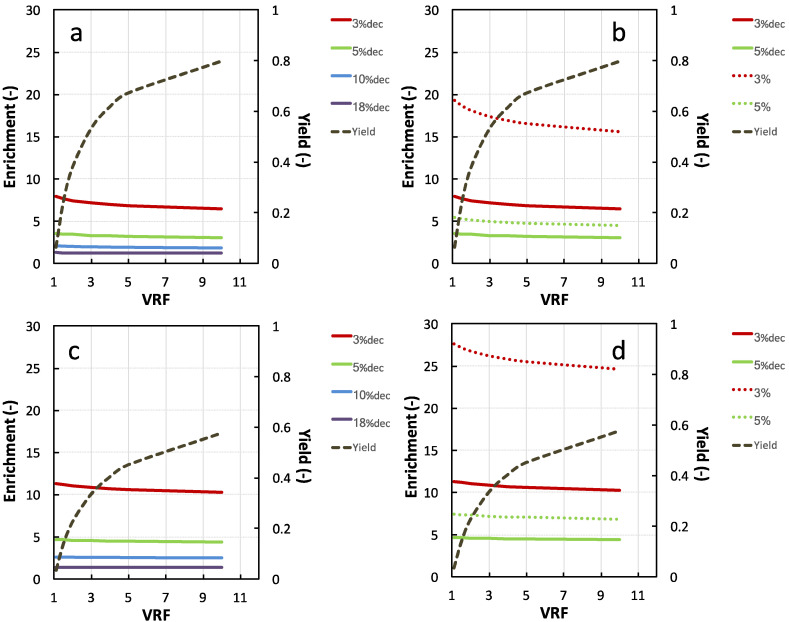
Calculated evolution of NKT yield and enrichment in the permeate compartment during the ultrafiltration of decolored 3%, 5%, 10% and 18%DH (**a**,**c**) and colored 3% and 5%DH (**b**,**d**) with the 3 kg·mol^−1^ membrane (**a**,**b**) and 1 kg·mol^−1^ membrane (**c**,**d**). Membrane calibration equations used for calculations were R= 0.7978 × log (MW) − 1.6206 and R= 1.0552 × log (MW) − 2.666 for 1 and 3 kg·mol^−1^, respectively.

Figure 3 shows the predictions of the evolution of the NKT yield and enrichment in the 1 and 3 kg·mol^−1^ membrane permeates for each hydrolysate. With 1 kg·mol^−1^ permeate, the highest hydrolysate DH were, the lowest NKT enrichments were. At the early stage of the UF (VRF = 1.1), the NKT enrichment values were 11.3, 4.6, 2.5 and 1.4 for decolored 3%, 5%, 10% and 18%DH_dec_, respectively. This is due to a higher proportion of peptides having a molecular size smaller than the membrane cut-off in the hydrolysates with higher DH. A larger fraction of peptides may, therefore, be transferred through the membrane with NKT. This was also observed in the reported work of Przybylski and coworkers [28] on NKT recovery by electrodialysis with ultrafiltration membranes (EDUF). The NKT enrichments remained constant during UF for 10% and 18%DH_dec_ while a decrease was observed for 5% and 3%DH_dec_. The largest enrichment values were observed for 3% and 5%DH_col_ (27 and 7.4 at VRF 1.1, respectively). For these two low DH hydrolysates, a decrease in NKT enrichment is also observed during the process.

With the 3 kg·mol^−1^ membrane, similar trends were observed, with lower enrichment values. The difference of NKT enrichment obtained with the two membranes was particularly marked with the low DH hydrolysates (with or without haem). Obviously, the strong discrepancies observed in NKT enrichments are due to the hydrolysate peptide molar weight distribution. Indeed, 18%DH hydrolysate contained 60% of peptides with a retention close to NKT (from 0.5 to 0.8 for 1 kg·mol^−1^ and 0.15 to 0.45 for 3 kg·mol^−1^ membrane). As comparison, 90% of 3%DH_col_ significantly had retentions higher than NKT (0.64 and 0.88 for 1 kg·mol^−1^ and 3 kg·mol^−1^). It can be noticed that the enrichment values obtained with 3%DH_col_ hydrolysate with both membranes (between 27 and 15) are outstanding for UF separation. As comparison, Przybylski and coworkers [28] observed no significant enrichment factor for the 3%DH hydrolysate separation by EDUF and a maximal enrichment factor of about 13-fold (the same analytical methods as for NKT quantification). Kapel et al. [21] observed an enrichment value in an antihypertensive peptide of only 1.3 upon UF of an alfalfa white protein hydrolysate with a 1 kg·mol^−1^ regenerated cellulose membrane at VRF 3.

The NKT yield in the 1 kg·mol^−1^ permeate compartment increased quickly up to VRF 5 (0.47) and raised then more slowly to reach 0.60 at VRF 10. The yields with 3 kg·mol^−1^ membrane followed the same pattern but showed higher values (0.65 at VRF 5 and 0.8 at VRF 10). As indicated by Equations (2) and (3), the peptide yield for a given VRF only depends on its retention value. Hence, the highest value of NKT retention with the 1 kg·mol^−1^ membrane than the 3 kg·mol^−1^ (0.652 vs. 0.305) is responsible for the lower yields.

On the one hand, NKT yield increases with FRV. However, on the other hand, NKT enrichment for the low DH hydrolysates decreases with FRV. From simulations, the 1 kg·mol^−1^ permeate of 3%DHcol hydrolysate would give the better enrichments. However, for a given FRV, the 3 kg·mol^−1^ membrane would show a far better yield. Besides, the 3 kg·mol^−1^ membrane would probably show a higher permeate flux. Hence, far better productivity should be expected from this last membrane. Since it is impossible to forecast the impact of the NKT enrichment or purity on the overall fraction antimicrobial activity, it was decided to implement the UF fractionation of this hydrolysate with both membranes and check the fraction bioactivity. A VRF 3 and 5 was chosen with the 3 and 1 kg·mol^−1^ membranes, respectively, to obtain the same yield value (near 50%).

It is important for industrial application that the enriched product is free of haem due to its unsuitable dark color. A high removal of haem in 3%DH_col_ in the permeate of each membrane should be expected, since a full haem retention was observed upon ultrafiltration of a bHb hydrolysate at low DH with a 10 kg·mol^−1^ regenerated cellulose membrane [32]. However, in the case of partial haem removal, 3%DH_dec_ was also included in the study.

### 3.3. Experimental Fractionation with 1 kg·mol^−1^ and 3 kg·mol^−1^ Membranes

**Figure 4:**
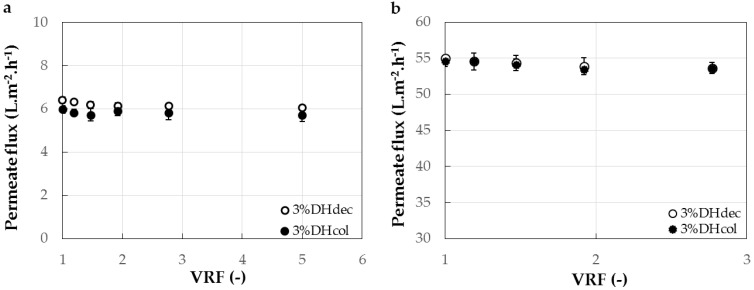
Evolution of the permeate flux during the ultrafiltration of decolored 3%DH_dec_ hydrolysate and colored 3%DH_col_ hydrolysate with the 1 kg·mol^−1^ (**a**) and 3 kg·mol^−1^ (**b**) membrane cut-offs (surface of 88 cm^2^) to a volume reduction factor (VRF) of 5 and 3, respectively.

Figure 4 shows the evolution of the permeate flux during UF with the 1 and 3 kg·mol^−1^ membranes of the 3% DH colored and decolored hydrolysates. The permeate fluxes observed were nine times superior with the 3 kg·mol^−1^ (Figure 4b) than with the 1 kg·mol^−1^ (Figure 4a) membrane. The figure also indicates that the flux remained rather stable during the fractionation. Eventually, it can be noticed that the presence of haem in the hydrolysate had no significant impact either on the permeate flux or the peptide transmission.

Table 1 shows the experimental NKT yields, purities and enrichments obtained during UF with the 1 and 3 kg·mol^−1^ membranes of the 3% DH colored and decolored hydrolysates. The experimental values were compared with the calculated values obtained from the simulation methodology. The fraction productivity (expressed in g·m^−2^·h^−1^) deduced from the process duration is also shown in this table. The table indicated that the relative errors between calculation and experiments were between 2 and 13%. This demonstrated the high accuracy of the simulation methodology described by Kapel et al. [21] with another type of hydrolysate.

**Figure 5:**
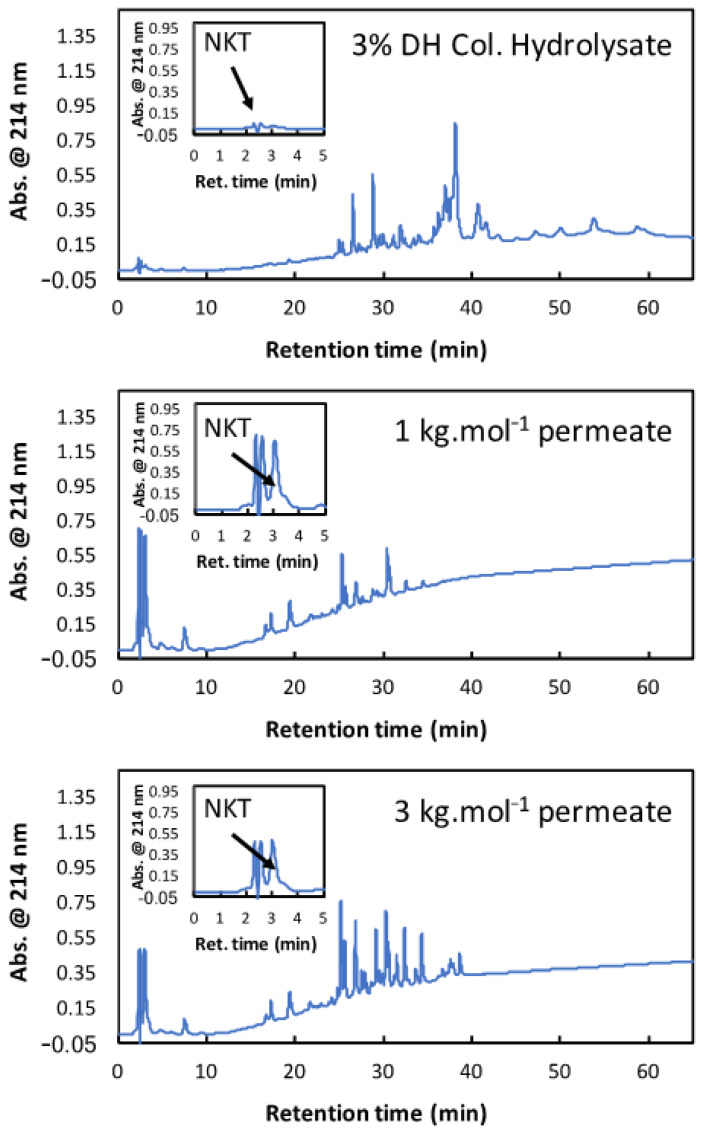
Reverse Phase-HPLC profiles of colored bovine hemoglobin hydrolysate at 3%DH and the permeate of this hydrolysate obtained with 1 kg·mol^−1^ and 3 kg·mol^−1^ membrane cut-offs.

Table 1 also shows that the experimental purities obtained with 3%DH_col_ and both membranes were two times higher than the purities obtained with 3%DH_dec_. For 3%DH_col_, remarkably high NKT purities were observed in the UF permeates (70.3% and 38.5% with the 1 and 3 kg·mol^−1^ membranes, respectively). It is very notable that such purity was reached using an ultrafiltration process with a similarly complex starting mixture. As a comparison, the best purity observed for the NKT selective separation by EDUF was 10.3% at 5%DH_dec_ [28]. In general, higher productivity was observed with 3%DH_dec_ as compared to 3%DH_col_. This is because the total peptide transmission was higher with this hydrolysate, while the permeate fluxes remained the same. However, the productivity observed with 3%DH_col_ and the 3 kg·mol^−1^ membrane (9.8 g. m^−2^·h^−1^) was higher than the reported transport rate value with EDUF implementation (0.67 g. m^−2^·h^−1^; [28]). These productivity values are also noteworthy for an ultrafiltration process of such a complex mixture. Taken as a whole, the UF of the 3%DH_col_ with both membranes offered great performances either regarding the NKT purity or the fraction productivity. Figure 5 shows the RP-HPLC profiles of this hydrolysate, the 1 kg·mol^−1^ permeate, and the 3 kg·mol^−1^ permeate. 3%DH_col_ showed an important peptide population containing high-molecular-weight peptides at retention times from 25 to 38 min. This population was globally hydrophobic. Some peptide peaks were observed from 3 to 20 min, representing small peptides that were less hydrophobic than those at high retention times. The peak corresponding to NKT was observed between 2 and 3 min and its weak area showed a low proportion in the total hydrolysate. The higher peak at 39 min was the haem. The undigested hemoglobin subunits were present from 40 to 60 min, meaning that the hemoglobin protein was not fully hydrolyzed by pepsin at this DH value. For the 1 kg·mol^−1^ permeate, the major peptide population was observed for retention times from 25 to 35 min and the undigested hemoglobin subunits were not present due to their high molecular weight (higher than 15 kDa). However, fewer peptides were present in this fraction compared to the whole hydrolysate due to the lower number of peaks. At around the 3 kg·mol^−1^ permeate, the same tendency was observed, but the peptide population was more important. Indeed, the number of peaks was more important, and their intensities were higher than with the 1 kg·mol^−1^ membrane. Furthermore, the NKT enrichment was better for the 1 kg·mol^−1^ permeate with a higher NKT peak observed at 3 min compared to the whole hydrolysate. Hence, the 1 kg·mol^−1^ permeate allowed a better NKT enrichment and purity, which is in accordance with the results presented above. Finally, the disappearance of the haem peak (39 min) on the RP-HPLC profiles of the 1 kg·mol^−1^ permeate and the 3 kg·mol^−1^ permeate proved that the 1 and 3 kg·mol^−1^ regenerated cellulose membranes can also retain the haem during the UF of bHb hydrolysates.

### 3.4. Evaluation of Peptide Fraction Antimicrobial Activity

The minimal inhibitory concentrations (µg_peptides_·mL^−1^) for the bHb hydrolysate (3%DH_col_) against the peptide fractions obtained in the permeate compartment after UF with the 1 and 3 kg·mol^−1^ membranes were presented in Table 2. The permeates obtained from the colored hydrolysate (3%DH_col_) with both membranes showed significant differences in terms of antimicrobial activities compared to the initial hydrolysate. This fact indicated that the antimicrobial activity of the peptide fractions was most efficient after UF separation. These results were in accordance with the NKT enrichment factors observed previously in the samples.

The 1 kg·mol^−1^ permeate obtained peptide fractions with a low MIC (under 20 µg_peptides_·mL^−1^ against all tested strains), indicating a strong antimicrobial activity for all tested strains compared to the initial hydrolysate (MIC values from 53.38 ± 15.1 µg_peptides_·mL^−1^ against *Escherichia coli* to 256.2 µg_peptides_·mL^−1^ against *Microccocus luteus*). Using the 3 kg·mol^−1^ MWCO membrane, the MIC values were higher than using the 1 kg·mol^−1^ MWCO membrane against *Microccocus luteus* (47.93 µg_peptides_.mL^−1^ for the 3 kg·mol^−1^ permeate and 2.06 for the 1 kg·mol^−1^) and *Listeria innocua* (39.94 ± 11.3 µg_peptides_·mL^−1^ and 16.43 µg_peptides_·mL^−1^ for the 1 kg·mol^−1^). The lower MIC obtained with the 1 kg·mol^−1^ MWCO membrane showed that fewer peptides were recovered in the permeate, corresponding to a better enrichment in NKT, as observed previously (28.7 against 15.7 with the 3 kg·mol^−1^ MWCO membrane). The MIC values remained in the same range against *Escherichia coli* (8.21 and 5.99 µg_peptides_.mL^−1^ for the 1 and 3 kg·mol^−1^, respectively) and *Salmonella enteretidis* (8.21 and 9.98 ± 1.50 µg_peptides_·mL^−1^ for the 1 and 3 kg·mol^−1^, respectively).

The most striking fact was that the 1 kg·mol^−1^ permeate from the 3%DH_col_ hydrolysate had an activity close to the standard NKT. From these results, it can be said that the antimicrobial activity was in relation with the process selectivity and the NKT purity. Indeed, using a 3 kg·mol^−1^ MWCO membrane showed peptide fractions that were less enriched in NKT with an antimicrobial activity that was less effective than when using a 1 kg·mol^−1^ MWCO membrane. This could be explained by the presence in the 3 kg·mol^−1^ permeate of more peptides (Figure 5), including in the group S1 of antimicrobial peptides [2]. This kind of peptide was less effective than the peptides of group S2 as NKT. Their presence decreased the NKT purity and, consequently, the bioactivity of the peptide fraction from UF permeates.

## 4. Conclusions

In this study, a simulation method was applied to predict the enrichment rate, extraction yield and purity of an antimicrobial peptide (NKT) in various bovine hemoglobin hydrolysates during ultrafiltration. It has been observed that the NKT enrichment decreased while the DH of the hydrolysate increased. From the prediction results, the most promising hydrolysates were identified (the colored and decolored 3%DH hydrolysates) and the experimental ultrafiltration conditions were rationally implemented for higher antimicrobial recovery. A remarkably high experimental NKT purity of about 70%, corresponding to an enrichment factor of about 29, compared to the initial hydrolysate, was obtained for the ultrafiltration of the colored 3%DH hydrolysate with the 1 kg·mol^−1^ MWCO-regenerated cellulose membrane at a VRF of 5. These results are noteworthy for a tangential ultrafiltration process of such a complex mixture. The permeate thus obtained showed excellent antimicrobial activity close to the standard NKT, demonstrating its promising potential use in food safety. Finally, the comparison of the experimental and simulated data demonstrated that the simulation methodology was valid with another case and proved its high reliability and utility.

## Figures and Tables

**Table 1:** Calculated NKT enrichment (-), yield (%), and purity (%) for colored and decolored 3%DH hydrolysates in the permeate compartment at VRF 3 and 5 for 3 and 1 kg·mol^−1^ membranes, respectively and experimental NKT enrichment (-), yield (%), purity (%) and fraction productivity (g.m^−2^·h^−1^). Experimental values are the means of two experimentations and relative errors between them were less than 5%.

Molecular Weight Cut-Off Membrane	*1 kg·mol^−1^ Permeate*		*3 kg·mol^−1^ Permeate*	
*Colored or decolored hydrolysate (3%DH)*	*Decolored*	*Colored*	*Decolored*	*Colored*
Initial NKT purity (%)	2.6	2.5	2.6	2.5
*Calc. NKT Enrich. (-)*	*10.7*	*25.5*	*7.2*	*17.4*
Exp. NKT Enrich. (-)	9.7	28.7	6.8	15.7
*Calc. NKT purity (%)*	*27.9*	*62.5*	*18.8*	*42.6*
Exp. NKT purity (%)	25.3	70.3	17.7	38.5
*Calc. NKT Yield. (%)*	*46*	*46*	*54*	*54*
Exp. NKT Yield. (%)	52	44	55	55
Exp. productivity	1.2	0.5	21.2	9.8
(g.m^−2^·h^−1^)				

**Table 2:** Minimal inhibitory concentrations (µg_peptides_.mL^−1^) for standard NKT and bovine hemoglobin hydrolysate (3%DH_col_) against peptide fractions obtained in the permeate compartment after ultrafiltration separation with 1 and 3 kg·mol^−1^ membrane cut-offs.

Sample	Colored or Decolored Hydrolysate (3%DH)	Molecular Weight Cut-off	Gram-Positive Bacteria		Gram-Negative Bacteria	
			*Microccocus luteus* ATCC 4698	*Listeria innocua* ATCC 33090	*Escherichia coli* ATCC 25922	*Salmonella enteretidis* ATCC 13076
NKT	Standard	/	5.85 *	0.65 *	5.85 *	3.25 *
Hydrolysate	Colored	/	256.2 ± 0.00	128.1 ± 0.00	53.38 ± 15.1	128.1 ± 0.00
Permeate	Colored	1 kg·mol^−1^	2.06 ± 0.00	16.43 ± 0.00	8.21 ± 0.00	8.21 ± 0.00
Permeate	Colored	3 kg·mol^−1^	47.93 ± 0.00	39.94 ± 11.3	5.99 ± 0.00	9.98 ± 1.50

* CMI values calculated from previous study [5] and expressed in µg peptides. mL^−1^.
